# Nurse clinic versus home delivery of evidence-based community leg ulcer care: A randomized health services trial

**DOI:** 10.1186/1472-6963-8-243

**Published:** 2008-11-26

**Authors:** Margaret B Harrison, Ian D Graham, Karen Lorimer, Elizabeth VandenKerkhof, Maureen Buchanan, Phil S Wells, Tim Brandys, Tadeusz Pierscianowski

**Affiliations:** 1 School of Nursing, Queen's University, 78 Barrie Street, Kingston, Ontario, Canada, K7L 3N6; 2 School of Nursing, University of Ottawa, 550 Cumberland Street, Ottawa, Ontario, Canada, K1N 6N5; 3 Ottawa Health Research Institute, Clinical Epidemiology Institute, 1053 Carling Avenue, Ottawa, Ontario, Canada, K1Y 4E9; 4 Carefor Health and Community Services, 1200 St. Laurent Blvd., Ottawa, Ontario, Canada, K1K 3B8; 5 University of Ottawa, The Ottawa Hospital Dept. of Medicine, 1053 Carling Avenue, Ottawa, Ontario, Canada, K1Y 4E9; 6 Ottawa Health Research Institute, 725 Parkdale Ave., Ottawa, Canada, K1Y 4E9; 7 Dept. of Surgery, 550 Cumberland St, Ottawa, Ontario, Canada, K1N 6N5; 8 University of Ottawa, 550 Cumberland Street, Ottawa, Ontario, Canada, K1N 6N5; 9 Dept of Medicine, University of Ottawa, 550 Cumberland Street, Ottawa, Ontario, Canada, K1N 6N5

## Abstract

**Background:**

International studies report that nurse clinics improve healing rates for the leg ulcer population. However, these studies did not necessarily deliver similar standards of care based on evidence in the treatment venues (home and clinic). A rigorous evaluation of home versus clinic care is required to determine healing rates with equivalent care and establish the acceptability of clinic-delivered care.

**Methods:**

Health Services RCT was conducted where mobile individuals were allocated to either home or nurse clinic for leg ulcer management. In both arms, care was delivered by specially trained nurses, following an evidence protocol. Primary outcome: 3-month healing rates. Secondary outcomes: durability of healing (recurrence), time free of ulcers, HRQL, satisfaction, resource use. Data were collected at base-line, every 3 months until healing occurred, with 1 year follow-up. Analysis was by intention to treat.

**Results:**

126 participants, 65 randomized to receive care in their homes, 61 to nurse-run clinics. No differences found between groups at baseline on socio-demographic, HRQL or clinical characteristics. mean age 69 years, 68% females, 84% English-speaking, half with previous episode of ulceration, 60% ulcers at inclusion < 5 cm^2 ^for < 6 months. No differences in 3-month healing rates: clinic 58.3% compared to home care at 56.7% (p = 0.5) or in secondary outcomes.

**Conclusion:**

Our findings indicate that organization of care not the setting where care is delivered influences healing rates. Key factors are a system that supports delivery of evidence-based recommendations with care being provided by a trained nursing team resulting in equivalent healing rates, HRQL whether care is delivered in the home or in a community nurse-led clinic.

**Trial registration:**

ClinicalTrials.gov Protocol Registration System: NCT00656383

## Background

Chronic wounds are not typically seen as a pressing health care problem, yet leg ulcers comprise a common, complex, and costly condition. International studies demonstrate that leg ulcer occurrence increases with age [1-5]; chronic leg ulcers are a significant burden to both patients and the health care system [6-10]. Patients tend to receive poorly integrated services in multiple settings. Delivery of leg ulcer care through integrated, nurse-led community clinics has been enthusiastically endorsed in the UK since the early 1990s [11-17]. It was postulated that not only would clinic-based care provide social support, encourage mobility, and improve patient outcomes through improved linkages and greater consistency of treatment between specialist and district home care, it would also reduce the number of providers and specialties involved. In the influential London Riverside project, five home nursing districts, one tertiary setting, and the Charing Cross Hospital were consolidated in a health services arrangement for clinic care provision [13,18]. A pre-post audit at 12 weeks revealed that leg ulcer healing rates improved from 22% to 69% with the new service. Subsequently two controlled studies [14,16], one of which was randomized [14,16], were unable to achieve such notable improvements as the Riverside project (24% to 34%, p = ns [14] and from 26% to 42%, p = 0.001 [16]).

Many countries are shifting to nurse delivered community care for the leg ulcer population despite a lack of comparison to equivalent home care.[19] On the surface, this is an appealing trend particularly from a resource perspective. Use of community clinics transfers the time and expense of travel to individuals from home care agencies, where the nurse travels to individuals' homes. Closer examination of these studies, however, indicates an inequity between clinic and home care groups; the implementation of nurse clinics are typically characterized by more skilled provider teams and closer adherence to evidence-based recommendations than the comparator groups which often neglected to include compression therapy. If the quality of provider teams, assessment, and management were held constant, would there still be improvement with care delivered in clinics?

Canada's unique challenges necessitated comparisons of clinic-based, community nursing care to home care models. Factors such as the weather extremes, distance to health services, variant urban-rural mix in many communities, and the limited experience with nurse-run clinics have to be considered. As well, the regional and individual acceptance of such a service delivery model is unknown.

To respond to this, we first undertook research to evaluate the impact of evidence-based leg ulcer care delivered with the usual model (home visiting) and clinics using a specially trained nursing team. In a one year pre-post evaluation we demonstrated improvements in 3-month healing rates from 23% to 56%, similar to rates achieved in the UK studies. Median supply costs declined from $1923 to $406 (Canadian dollars) and nursing visits reduced from 37 to 25 visits per case [20]. In the post-implementation phase, we nested a randomized controlled trial into the design to determine the relative effectiveness and efficiency of clinic-delivered versus home-delivered care with individuals who were mobile. Both the control (home care) and experimental (clinic) arms were treated by the same team of specially trained nurses, and both followed the same evidence-based protocol for care including compression bandaging [21]. The intent of the trial, within our larger effort of implementing evidence-based wound care, was to rigorously evaluate the setting of care in order to inform reorganization efforts as to whether community home care, clinics, or both could be options for Canadian home care authorities. The resource utilization information, albeit limited to data available from administrative sources is intended to provide foundational information for further study. This paper reports the results of that randomized trial of the effectiveness and efficiency of home versus clinic delivered care for individuals with leg ulcers.

## Methods

### Design

We conducted a prospective, randomized two-arm trial to evaluate the effectiveness and efficiency of community leg ulcer clinics (intervention) with care delivered through home visiting (usual care) by comparing individual and system level outcomes, including a one year post-healing follow-up. We hypothesized that receiving care in the clinic setting would improve healing rates at 3 months (primary outcome).

### Setting and sample

From a large urban-rural region in Ontario overseen by two Community Care Access Centres (home care authorities) and two home nurse agencies, the study population was comprised of those eligible for community leg ulcer care, including those already in care and any new referrals who met four inclusion criteria:

1. Admission to home care for care of a leg ulcer (an ulcer below the knee to the foot)

2. Having leg ulcer of venous or mixed venous and arterial etiology and eligible for compression bandaging.

3. Ability to travel to clinic (i.e. individuals able to travel outside their home)

4. No major contraindication for clinic care (i.e. domestic issues that would decrease compliance for clinic visits e.g. not being able to leave an ill spouse)

### Procedures

Upon referral to the regional home care service for leg ulcer care, individuals received a comprehensive, standardized clinical assessment by specially trained registered nurses. Mobile individuals, i.e. either independently mobile or requiring minimal assistance, were informed of the study and invited to participate. Consenting individuals were then randomly allocated to receive leg ulcer care in either a nurse-run clinic or in their own home. A computer-generated schedule of randomization was used to allocate participants to community clinic or home care. Allocation was sealed in opaque, serially numbered envelopes and to eliminate any possibility of bias, randomization was controlled centrally from the university research office and stratified by client status (existing client or new referral).

The same nursing team delivered care in both the home and clinic settings. Care for leg ulcer management was standardized and guided by international evidence-based recommendations [12,21]. The leg ulcer care management protocol was developed using the Practice Guideline Evaluation and Adaptation Cycle (PGEAC) which is a process for evaluating and adapting existing practice guideline recommendations for local use [21-23]. The process involved bringing together an interdisciplinary task force comprised of clinical leaders representing home nursing, enterostomal therapy, family practice, vascular surgery, dermatology, and haematology to systematically identify and review existing leg ulcer guidelines. The task force appraised the quality of the existing recommendations using the Guidelines Appraisal Instrument [24,25], their content, clinical utility, and feasibility of implementing the recommendations in the local context and produced a local care protocol adapted from the guidelines reviewed [22]. We sent the draft protocol to home care nurses and family physicians for review and feedback [26,27]. It was kept current through scheduled reviews by the task force [28]. Agency nurses involved in the study received additional training and were familiar with the evidence for practice supporting the guideline recommendations.

#### Ethics, consent and random assignment to setting of care

Home care decision-makers weighed the potential risks and benefits of randomly assigning clients to *setting *of care and concluded that the potential benefits outweighed the risks because: 1) the duration of the study was limited, 2) flexibility was built into the study design to permit clinicians to exclude clients from clinic care if clinical or social circumstances warranted it and these would be tracked, and 3) the need for robust findings was essential to determine the value of providing leg ulcer clinic care in Canada.

Prior to their first visit, all clients meeting the criteria for clinic care were provided with a written explanation about the need and rationale for the study. At the time of intake assessment, an attending nurse verbally explained the trial and sought written informed consent. Assistance with transportation to the clinic was available in the form of taxi chits or bus tickets if required. Ethics approval was received from the Ottawa Health Research Institute Ethics Board.

#### Data collection and management

Baseline data collection began at the time of initial assessment through interview, clinical assessment and chart review. Socio-demographic and clinical assessment, and measurements of primary and secondary outcomes were collected at baseline. Measurement of ulcer size was repeated at 3-month intervals until complete healing, or until 12 months post study entry, whichever came first. If healing occurred between these intervals measurements were taken at that time.

Quality assurance procedures ensured the integrity of the trial [29-31]. Aspects of data management were elaborated in a detailed protocol manual for the study team. A log record was maintained to track the status of participants throughout the duration of the trial. Once recruited, participants were assigned a code number used on all subsequent documentation to ensure confidentiality. Withdrawals from either arm of the study were monitored and reasons documented. Data were analyzed using SPSS (version 12) software. Case records (10%) were randomly selected to assess data entry accuracy every 3 months.

#### Outcome measurement

The primary outcome for the trial was healing at 3 months. Other healing outcomes were reduction in size and sustainability of healing. These measures were chosen based on clinical feasibility and to allow comparisons to other leg ulcer studies in the literature. 'Healed' was defined as re-epithelialisation of all ulcers [14]. The 3 month timeframe to healing continues to be used in many studies [32] and is encouraged by international practice guidelines as it serves as a useful quality outcome [33,34]. Ulcer size was assessed by calculating ulcer area (cm2). The line of epithelium was traced on acetate with an indelible pen and the ulcer area calculated using computer planimetry. This method to serially measure ulcer size has been shown to be reliable and valid [33,35-37]. As with our previous studies, attending nurses are alerted to report to the trial office (phone number is on chart) when an ulcer has healed so that the date of healing can be noted in the tracking database. This is typically the point when individuals come off of service and if there was any concerns a further visit was set-up with the clinical leader of the service. It was not possible to conduct blinded outcome assessment at the point of care given the provider would already know which setting care was being received. Additional independent, blinded visits at a separate location, given the dispersal of individuals in the community would have been prohibitive. Sustainability of healing was assessed by the time to first recurrence after the ulcer healed, and time free of ulcers during the 12-month follow-up from admission.

Pain and health related quality of life were assessed using the McGill Short Form Pain Questionnaire (SF-MPQ) [38-40], and the Medial Outcomes Study Short-Form Health Survey [41,42]. The SF-MPQ is designed to assess the multi-dimensional nature of the pain experience and has been demonstrated to be a reliable, valid and consistent measure, and it has been used in studies of patients with leg ulcers [14,43,44]. The Short Form Health Survey measures self-reported aspects of HRQOL, including physical function, role physical, bodily pain, general health, vitality, social function, role emotional and mental health. Two component scores, the Physical and Mental Component Summaries, are standardized to a mean of 50, with a score above 50 representing better than average function and below 50 poorer than average. The pain and HRQL instruments were selected based on our previous work[43,44] and that of Walters et al. [10,45]. In the Walters study, the SF-36, EuroQol (EQ), SF-MPQ and the Frenchay Activities Index (FAI) were evaluated with 233 individuals on three occasions over one year. The SF-MPQ was more responsive than the other instruments and detected changes at the 3-month and 12-month follow-up. Based on these results it was recommended that SF-MPQ be used to evaluate outcomes of interventions with a short-term follow-up (three months), and that SF-36 be used for 12-month follow-up with or without the SF-MPQ. From the SF-36 a shorter 12-question version (SF-12) has been developed and evaluated [41,46] with the Physical and Mental Component Scores correlating very highly with the SF-36 version (Medical Outcomes Trust source pages). Thus we opted to use the shorter SF-12 to reduce the burden of response as we have found in previous studies it took 5–8 minutes to complete compared to 11 minutes for the SF-36. Canadian normative data were used for comparison purposes with the SF-12 [47].

Satisfaction with care was assessed through two surveys administered at the 3-month point. This provided data on individual's perceptions of the impact of the leg ulcer on their personal lives and the satisfaction with the care they received in either the clinic or home setting.

Basic resource use for an episode of ulcer care (referral to healing) related to the both clinic and home setting were assessed including; number and weekly frequency of visits, all supplies and materials provided through the home care authority. The Practice Audit Tool, used in our previous work, captures supplies (bandaging and dressing) and nursing visits for the clinic or home care. Acquisition costs of the different bandage systems and other supplies was calculated from the retail prices quoted by the manufacturers. Expenditures differ depending on setting of care for both the system and the patients and families. Given this a comprehensive economic analysis would require a *societal*, rather than sector, perspective. In such an analyses, all expenditures such as travel time to provide or receive care (nurse and patient), vehicle allowances for nurses, overhead of the community located leg ulcer clinic settings (rent, utilities, reception, etc.), and family expenditures related to leg ulcer care (time off work, travel, out-of-pocket expenses etc.) would be required. It was beyond the scope of this trial to assess all the indirect and direct costs related to home and clinic care. Basic resource data on an episode of care (admission to discharge) collected in this trial may provide the basis in planning for a full economic analysis or modeling study.

#### Sample size

From data collected in our initial prevalence study, 20% of cases had healed within a 3-month period, comparable to rates of ulcer healing reported in the literature. Thus sample size was based on detecting a difference of 20% in 3-month healing rates in favour of the clinic setting. We expected the absolute rate of healing in the clinic group at 3-months to be 40%, a figure falling between the extremes (20%–89%) for 3-month healing rates in previously reported leg ulcer clinic studies [11,13]. To detect a 20% difference, with a power of 80% at a significance level of .05 (two-tailed), 100 patients per group was required.

#### Analyses

The primary data analysis was based on 'intention to treat'. The proportion of individuals in each group healed at 3 months (91 days) and recurrence rates were compared using Chi squared tests. Mean number of visits for the ulcer episode (admission to discharge), weeks on service, visits per week were calculated for the home and the clinic groups. Mean differences in health status outcomes (SF-12, pain) and resource variables were compared between the treatment arms of the trial using the independent t-test of either the pooled or separate variance estimates as appropriate. Variables with a non-normal distribution were analyzed with the appropriate non-parametric procedures, Mann-Whitney for unpaired data and Wilcoxon for paired data.

## Results

**Figure 1:**
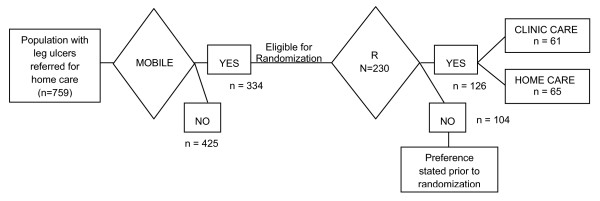
Flow of population with leg ulcers and eligible pool for clinic vs home trial.

Over a 37 month recruitment period (November 7 2000 – March 17 2003), 759 individuals referred for home care were screened (see Figure 1). Of these 44% (n = 334) were assessed as mobile and able to travel outside their homes, and therefore eligible to receive clinic care. After assessment, 69% (n = 230) presented with venous disease or mixed venous and arterial etiology and were eligible for compression bandages. When approached, 45% (n = 104) indicated that they were willing to be studied in terms of healing but declared a *strong *preference for receiving care in either the home or a clinic setting. Therefore, this group was followed but not randomized. The remaining 126 people were willing to be randomized and these individuals were entered in the trial. There were no significant differences on profile characteristics between the 'stated preference' group and the trial group.

Of the 126 trial participants, 65 were randomized to receive care in their homes, and 61 to the nurse-run clinic. There were no differences on admission between the participants randomized to either the clinic and home care groups on their socio-demographic, circumstance of living, health-related quality of life or clinical characteristics (Table 1). Mean age of the trial group was 69 years. There were more women (68%) than men and the majority was English-speaking (84%). Half had at least one previous episode of ulceration and 60% experienced a current ulcer smaller than 5 cm^2 ^for less than 6 months. Health related quality of life was poor on the SF-12 physical component; these individuals' baseline scores were much lower than the Canadian norm (35.1 vs 51.7). The SF-12 mental component score was similar to the Canadian norm (49.7 vs 50.5).[47] In tracking the evidence-based protocol, there were no differences found in key aspects of the clinical care received by the two groups (Table 2).

**Table 1:** Comparison of the baseline characteristics of the study population and those allocated to clinic care and home care groups

	TOTAL (n = 126)	Home Group (n = 65)	Clinic Group (n = 61)	p-value
	n(%)	n (%)	n (%)	
Etiology of leg Ulcer				
• Venous	86 (68.3)	45 (69.2)	41 (67.2)	0.85
• Mixed	40 (31.7)	20 (30.8)	20 (32.8)	
Gender-Female	71 (56.3)	35 (53.8)	36 (59.0)	0.59
Language-English	106 (84.1)	56 (86.2)	50 (82.0)	0.79
Living Alone	54 (42.9)	29 (44.6)	25 (41.0)	0.72
Independently Mobile	89 (70.6)	45 (70.3)	44 (72.1)	0.84
Previous Ulceration	62 (49.2)	34 (52.3)	28 (45.9)	0.48
Prognostic Indicators				
• Duration < 6 months, Ulcer size < 5 cm^2^	76 (60.3)	39(60.0)	37 (60.7)	0.13
• Duration > 6 months, Ulcer size < 5 cm^2^	13 (10.3)	5 (7.7)	8 (13.3)	
• Duration < 6 months, Ulcer size > 5 cm^2^	23 (18.3)	10 (15.4)	13 (21.7)	
• Duration > 6 months, Ulcer size > 5 cm^2^	14 (11.1)	11 (16.9)	3 (5.0)	
Ulcer Size (cm ^2^) (median, range)	2.25 (.01–116.6)	2.4 (.04 – 116.6)	2.3 (.01–84.0)	
Continuous variables	Mean (SD)	Mean (SD)	Mean (SD	
Mean Age (years)	68.5 (14.1)	68.3 (15.3)	68.7 (12.9)	0.86
SF12 Scores		(n = 58)^1^	(n = 52)^1^	
• Mental Component	49.7 (11.0)	50.9 (10.8)	48.4 (11.2)	0.75
• Physical component	35.1 (9.9)	35.5(10.3)	34.7 (9.7)	0.43
Diathesis in years	14.2 (15.9) Median 8.0	11.0 (13.8) Median 7.0	18.2 (17.7) Median 10.0	0.05
Duration at initial assessment in weeks	30.4 (65.0)	24.98 (43.65)	36.26 (81.87)	0.96

^1 ^12.7% of the clients were unable because of language barrier or unwilling to complete the Quality of Life questionnaires

**Table 2:** Description of the evidence-based protocol delivered in the clinic care and home care groups

Variables	Home Group (n = 65)	Clinic Group (n = 61)	p-value
	% (n)	% (n)	
Comprehensive Clinical Assessment	100 (65)	100 (61)	
Doppler ABPI	96.9 (63)	96.7 (59)	.970
Compression Therapy			
All*	95.4 (62)	93.3 (56)	.709
• Venous disease*	53.7 (44)	46.3 (38)	.599
• Mixed Disease*	50.0 (18)	50.0 (18)	1.0

*Fisher's Exact Sig. (2-sided)

### Healing

The healing rate was not significantly different between groups. 58.3% of the group receiving clinic care healed at 3 months compared to 56.7% for those receiving home care (p = 0.5). Similarly there was no significant difference in durability of healing; recurrence rates within one year were 24.6% in the clinic group compared to 21.5% (p = 0.42) in the home group (see Table 3). Mean time free of ulcers after healing was 190 days (sd 91.5 days) for the home group and 159 days (sd 87.3 days) for those receiving care in the clinic. Five cases were missing in the home group and one in the clinic group on the primary outcome due to refusal, unable to track or deaths.

**Table 3:** Healing, quality of life, and pain outcomes for those receiving an evidence-based protocol for leg ulcer care in either a nurse clinic or home care setting

Outcome	Home Group	Clinic Group	p-value
Healing	(n = 60)^1^ % (n)	(n = 60)^2^ % (n)	
• 3-month (91 days)	56.7 (34)	58.3 (35)	0.50
Recurrence rate in one year	21.5 (14)	24.6 (15)	0.422
Pain	(n = 52)^3^	(n = 49)^4^	
• No pain	51.9 (27)	63.3 (31)	0.496
• Mild/Discomfort	36.5 (19)	26.5 (13)	
• Distressing/horrible/excruciating	11.5 (6)	10.2 (5)	
SF12 Scores	(n = 52)^3^ n (sd)	(n = 46)^5^ n (sd)	
• Mental Component	51.8 (11.8)	53.9 (9.1)	0.33
• Physical Component	39.2 (11.9)	38.9 (10.7)	0.89

^1^missing 5 cases

^2^missing 1 cases

^3^missing 9 cases

^4^missing 13 cases

^5^missing 15 cases

Note on missing cases: at the 3-month point cases are missing on the survey data (SF-12 and pain) due to individuals not being willing to complete forms. They agreed to have their clinical data collected but were not willing to complete any forms.

### Pain and health-related quality of life

At 3 months, the majority of the clinic group (63%) reported 'no pain' compared to 52% of the home care group with no statistically significant differences between the groups on their pain assessments. Health related quality of life scores physical (PCS) and mental component scores (MCS) were not statistically different between the groups. In comparison to baseline, the clinic group improved more on the MCS (from 48.4 to 53.9) than the home care group (50.9 to 51.8).

### Individual's satisfaction with care

Overall the majority of individuals were very satisfied (95%) with the care received in the past 12 months and 93% would recommend it to others (Table 4). Individuals receiving clinic care reported less waiting time for nurse with 80% of the home having 30 minutes or less compared to 98% of clinic group (p = 0.03). Both home and clinic groups reported high satisfaction levels with the information they receive on how to care for their leg ulcers (98% vs. 96% very/quite satisfied, respectively). Similar proportions in both the home and clinic groups reported continued difficulties with normal activities (47% and 56% respectively) with the majority not feeling anxious or depressed (72% and 73%).

**Table 4:** Comparison at 3 month of the individuals perception of personal issues related to leg ulcer and satisfaction with care between those allocated to clinic care and home care groups

	Home Group (n = 54)	Clinic Group (n = 48)	p value
	% (n)	n (%)	
ISSUES (n = 102)			
Some problems walking about	59.3 (32)	45.8 (22)	0.23
Some problems with washing, dressing self	18.5 (10)	12.5 (6)	0.43
Some problems performing my usual activities	42.6 (23)	56.3 (27)	0.28
Not anxious or depressed	72.2 (39)	72.9 (35)	0.59
SATISFACTION (n = 97)	(n = 51)	(n = 46)	
• Waiting less than 30 minute	80.4 (41)	97.8 (45)	0.03
• Waiting 30 minutes – 1 hour	15.7 (8)	2.2 (1)	
• Waiting 1–2 hours	3.9 (2)	0 (0)	
Very/quite Satisfied with information	98.0 (50)	95.6 (44)	0.25
Very/quite Satisfied with treatment last 12 weeks	94.0 (47)	93.2 (41)	0.70
Very/quite Satisfied with nurses' skill	94.1 (48)	95.5 (43)	0.49
Recommend/highly recommend care you receive to others	93.9 (46)	90.9 (40)	0.58

### Resource use

There were no differences statistically in the groups on total number of nursing visits or visits per week, weeks on service, or supplies (Table 5). On average 37 visits were incurred for the episode of leg ulcer care (until healed) with the average weeks on service being 18.7 for home care and 17.3 for clinic care. It was not feasible to track the actual time of visits used in the clinic and in home. Although the number episodes of care were similar, this does not account for additional travel time incurred by nurses delivering home care. Due to constraints in how much data individual nurse could collect we do not have accurate information on the travel times for the home delivery arm. This initial data will allow for economic modeling in a further study.

**Table 5:** Resource utilization and costs by study group

Variables	Home (n = 65)		Clinic (n = 61)		Statistics
	Mean	(sd)	Mean	(sd)	p value
Nursing Visits for episode of Leg Ulcer Care	37.61	(31.596)	37.18	(44.328)	.950
Weeks on Service	18.66	(14.453)	17.30	(14.659)	.602
Visits per Week	2.12	(1.158)	2.12	(1.125)	.950
Nursing Cost for Episode of Care of Leg Ulcer Client	$1,868.01	($1,615.24)	$1,807.79	($2,163.87)	.859
Cost of Wound Supplies for Episode of Care	$1,137.39	($1,516.93)	$905.14	($1,714.91)	.423

## Discussion

In this community trial, people with leg ulcers who were mobile were randomized to receive care in either their homes or in a nurse-led clinic. Care was delivered by the same specially trained team of nurses in both settings guided by an evidence-based protocol for assessment and management. Healing at 3-months and recurrence were comparable and not statistically different between the clinic (58.3% healed, 24.6% recurred) and home care group (56.7%, 21.5% recurred). Both groups had relatively high healing rates at 3 months compared to what has been reported in the literature which is likely due to the organization and consistency in delivery of evidence-based care in both settings.

In our first study[20] using a pre-post design, we demonstrated the feasibility and acceptance of using an evidence-based protocol. Results indicated that the implementation of evidence-based care with a specially trained team was related to a more than doubling of healing at 3-months and savings in terms of nursing time and supplies. Further, this randomized health services trial provides the first comprehensive evaluation of clinic versus home care in a Canadian context. Unlike the UK studies, we found similar outcomes in healing regardless of venue of care provided that the quality of care is similar. This is due to the organization of care in home and clinic being similar in the current study whereas, the home delivered care was more varied in the previous trials. We conclude that organization of care is more important than setting with the key elements being: an explicit evidence-based protocol, specially trained nurses with enough wound practice to maintain skills, accessible equipment (e.g. dopplers, high compression bandages), and administrative support for a longer first visits to complete a comprehensive initial assessment and development of a care plan. Given this, health service planners and decision-makers could offer community leg ulcer care in either a clinic or home setting and expect good results if the team follows an evidence protocol and are specially trained. Such an option is important in the Canadian environment because factors such as, the size of the population, urban-rural mix, as well as feasibility to set-up clinics in a region is quite variable. Practically speaking, clinic care has the advantage of readily available supplies that may hasten immediate implementation of care once the comprehensive assessment is complete compared to the home setting where supplies may have to be ordered and delivered, resulting in a delay in implementation of the treatment plan. With nursing hours typically considered a scarce resource and with increasing pressure on home care resources to take on more, authorities might consider offering able clients the option to attend clinics to access care.

An interesting phenomenon discovered during this trial was that, although there was enthusiasm to participate in a wound study with one-year follow-up commitment, many participants expressed strong preferences with 54% of these preferring home delivered care and 46% preferring clinic delivered care. In total, 40% of potential trial participants were excluded from randomization due to stated preference. A future study will examine if after adjustment for co-morbid conditions, and severity and duration of ulcer, whether populations with stated treatment preferences have better outcomes regardless of treatment selected.

### Limitations of the study

Our trial has some limitations. First, it might be considered a limitation that nurses providing care were involved in the collection of data. Blinding the nurse to the setting was obviously not feasible and once bandages were applied it would have been excessively intrusive (and monumentally expensive) to remove them solely for the purpose of an outcome assessment. On the other hand, our small team of dedicated specially trained nurses, ensured expert, quality outcome assessments by measuring healing in a rigorous and consistent manner regardless of setting.

Secondly, due to an unexpected expression of strong preference for either clinic or home-delivered care the trial resulted in being underpowered, however given the observed difference in rate of healing between the two arms (1.6%). Assuming this difference is clinically significant (which most would agree it is not), the trial would have required a sample size of approximately 15,000 per arm to detect this difference. Even if the current trial continued until the predicted sample size was achieved (100 per arm) and the remaining ~40 patients per arm that were recruited during this period, demonstrated a 20% rate of healing in the clinic and a 40% rate of healing in the home, the final healing rates of ~52% for clinic and ~45% for home, would not be statistically significant. The 1.6% difference obtained in our study is nowhere near the 20% difference anticipated based on previous studies. Further, if this difference was considered clinically important, which arguably it is not, we would have needed ~800/arm to demonstrate it. So although sample size was not achieved, we were able to demonstrate that there were no significant differences in healing rates between the two groups and it will hopefully provide data for others to use in developing sample sizes for future wound studies.

Thirdly, patient preference may have an impact on the type of health services provided in a geographical area. It will be important for future studies to systematically monitor the reasons for an individual's stated preference to be treated in either the home or at a clinic. In general, comments indicated that many patients preferred clinic because it gave them a specific appointment time versus not knowing when the nurse was going to arrive at their home, particularly for employed patients. For some, the clinic provided an outing with social contact that would otherwise not be available to them, while others felt it too difficult to make the arrangements required to visit the clinic, including transportation, parking and distance to walk. However, many of the factors that lead to a stated preference (e.g., mobility, employment, social contact) also introduce bias into the sample, therefore future studies are necessary to examine the impact of preference on outcome. Finally, our study took place in two regions within southeastern Ontario that included both urban and rural components, and a culturally mixed population. These populations may not be representative of other areas where high density or more homogenous populations are found.

## Conclusion

The results from this health services trial differ from previous reports which have concluded that clinic care is superior to home care at improving leg ulcer healing. Organization of care, rather than setting is the crucial factor. We found that when quality wound care was supported with a service model, i.e. following evidence-based recommendations, and being delivered by a well-trained, dedicated nursing team, that similar healing results can be attained whether the leg ulcer care is delivered in the home or in a nurse clinic. Our data also suggests that a significant proportion of patients have preferences for care in both settings. The trial provides the first Canadian data on this health services issue and from a planning perspective, if feasible in a region, health authorities should consider both settings in order to provide mobile individuals with choice. Future research will be needed to investigate the relative efficiencies of clinic care compared to usual home care with particular attention to local environment, the various economies of scale (large, medium and small health regions), population dispersion between urban and rural, numbers of nursing agencies and nurse providers contracted to deliver wound care, organization of primary care and referral patterns and other important contextual factors.

## Competing interests

The authors declare that they have no competing interests.

## Authors' contributions

MBH and IDG were principal investigators on the trial and responsible for conceptualization, ethical approval, the conduct and management of the RCT and interpretation of the data.

KL, EVK, MB, PW, TB and TP comprised the trial co-investigators team and contributed to the design of the study, analyses and interpretation of data.

All authors have read and approved the final manuscript.

## Pre-publication history

The pre-publication history for this paper can be accessed here:


